# Variation of Soil Organic Carbon and Its Major Constraints in East Central Asia

**DOI:** 10.1371/journal.pone.0150709

**Published:** 2016-03-02

**Authors:** Xinqing Lee, Yimin Huang, Daikuan Huang, Lu Hu, Zhaodong Feng, Jianzhong Cheng, Bing Wang, Jian Ni, Tserenpil Shurkhuu

**Affiliations:** 1 State Key Laboratory of Environmental Geochemistry, Institute of Geochemistry, Chinese Academy of Sciences, 99 Lincheng West Road, Guiyang, 550081, Guizhou, China; 2 Key Laboratory of West China's Environmental System (Ministry of Education), Lanzhou University, 298 Tianshui Road, Lanzhou, 730000, Gansu, China; 3 Nuclear Research Center, National University of Mongolia, 14200, Ulaanbaatar, Mongolia; Tennessee State University, UNITED STATES

## Abstract

Variation of soil organic carbon (SOC) and its major constraints in large spatial scale are critical for estimating global SOC inventory and projecting its future at environmental changes. By analyzing SOC and its environment at 210 sites in uncultivated land along a 3020km latitudinal transect in East Central Asia, we examined the effect of environmental factors on the dynamics of SOC. We found that SOC changes dramatically with the difference as high as 5 times in north China and 17 times in Mongolia. Regardless, C:N remains consistent about 12. Path analysis indicated that temperature is the dominant factor in the variation of SOC with a direct effect much higher than the indirect one, the former breaks SOC down the year round while the latter results in its growth mainly via precipitation in the winter half year. Precipitation helps accumulate SOC, a large part of the effect, however, is taken via temperature. NH_4_^+^-N and topography also affect SOC, their roles are played primarily via climatic factors. pH correlates significantly with SOC, the effect, however, is taken only in the winter months, contributing to the decay of SOC primarily via temperature. These factors explained as much as 79% of SOC variations, especially in the summer months, representing the major constraints on the SOC stock. Soil texture gets increasingly fine southward, it does not, however, constitute an apparent factor. Our results suggested that recent global warming should have been adversely affecting SOC stock in the mid-latitude as temperature dominates other factors as the constraint.

## Introduction

Soil organic carbon (SOC) is the largest carbon stock in the terrestrial ecosystem. A small change of it would have a large impact on the atmospheric CO_2_ [1]. Recent global warming raised a great concern over the carbon stock at the change of climate and other environmental factors. Despite many studies, consensus still remains to be reached. While some studies indicated no effect of temperature on SOC [2, 3], others observed accelerated decomposition with temperature going up [4–7], especially in the high-latitude [8, 9]. Likewise, the effect of precipitation is also controversial with some reporting precipitation conducive to SOC accumulation [10, 11] while others not [12]. Similar debates also involved other environmental factors such as soil type, texture, pH, N content, vegetation type and land use forms [13, 14]. These contradictions may derive from the studies involving relatively small areas or limited number of sites since the apparent constraints of SOC may change in space [15]. The results can be misleading when extrapolated to large spatial scale or confounding in projecting the future of SOC stock [16]. Given the estimation of SOC inventory and its future projection often involve scales as large as the continental or global, it is helpful to understand the dynamics of SOC in consistent scales. Furthermore, large scale often creates some environmental factors changing so dramatically that they may dwarf many other local or even sub-regional ones, facilitating isolation of their effect. Despite these advantages, large-scale-based studies are few and far between [17–21]. Eurasia continent is the largest terrestrial ecosystem of the world, the environment changes greatly, especially in the south-north direction, and thus provides an ideal background for studying SOC in large scale. We examined the dynamics of SOC in the east part of the continent, including north China and whole Mongolia. Here we report our findings.

## Materials and Methods

### Geographic and Climatic Background

**Fig 1:**
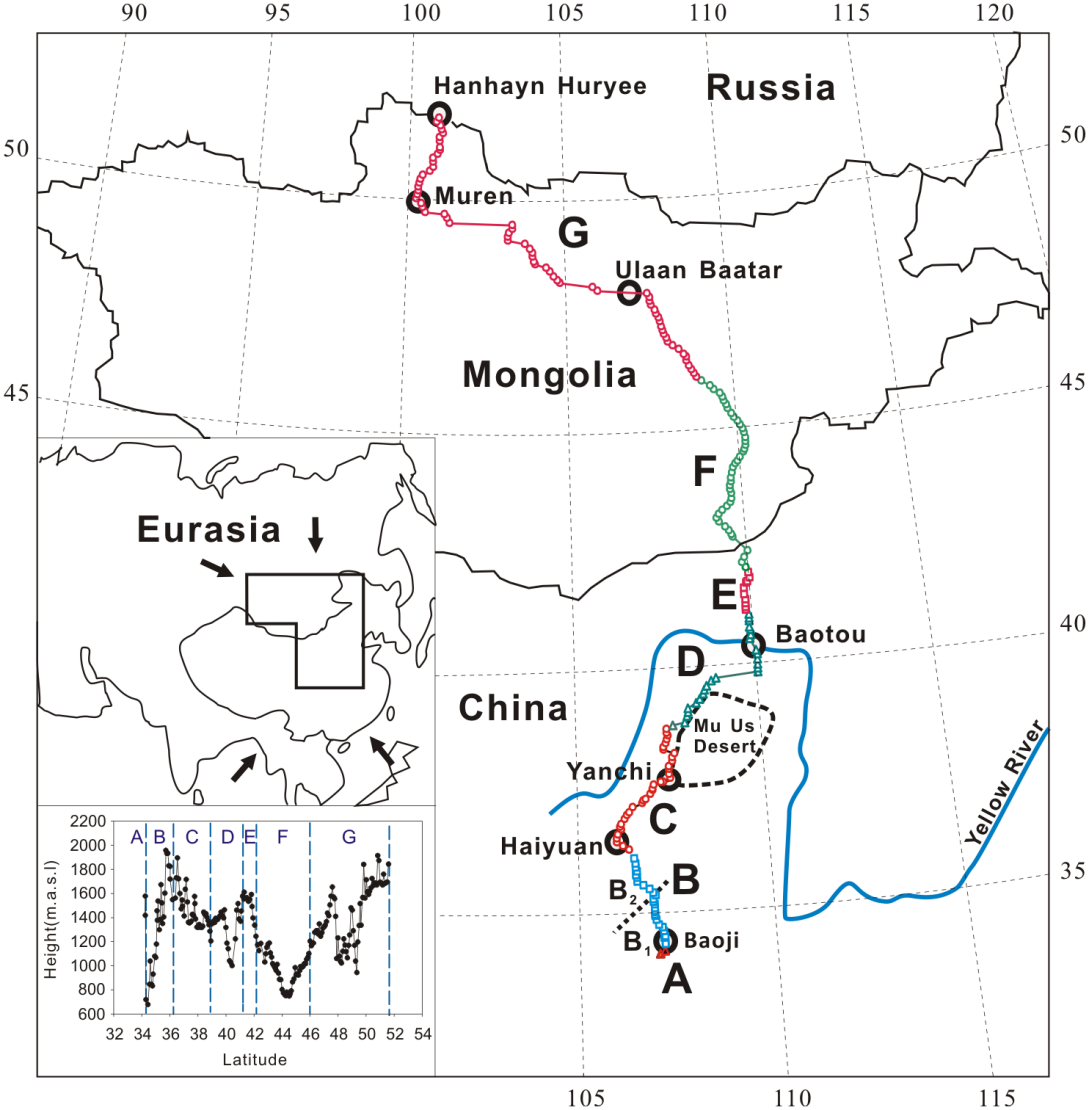
Geographic background of East Central Asia and division of the sampling transect. A–northern slope of the Qinling Mountains, central China; B–Loess Plateau, north China, which is dichotomy between the south (B_1_) and north (B_2_); C—central and east Ningxia Autonomous Region and southwest edge of the Mu Us sandy land, north China; D—northwest Ordos plateau, Yellow River valley and the southern slope of Yinshan mountains, north China; E—northern slope of the Yinshan Mountains, north China; F—Gobi desert in north China and south Mongolia; G—north Mongolia. The dots, circles, squares and triangles in various colors in the transect indicate the sampling sites. Insert above: study area in the Eurasia continent, the white arrows indicate the advancing directions of maritime air-masses; Insert below: elevation changes in the transect in meters above sea level (a.s.l.).

**Fig 2:**
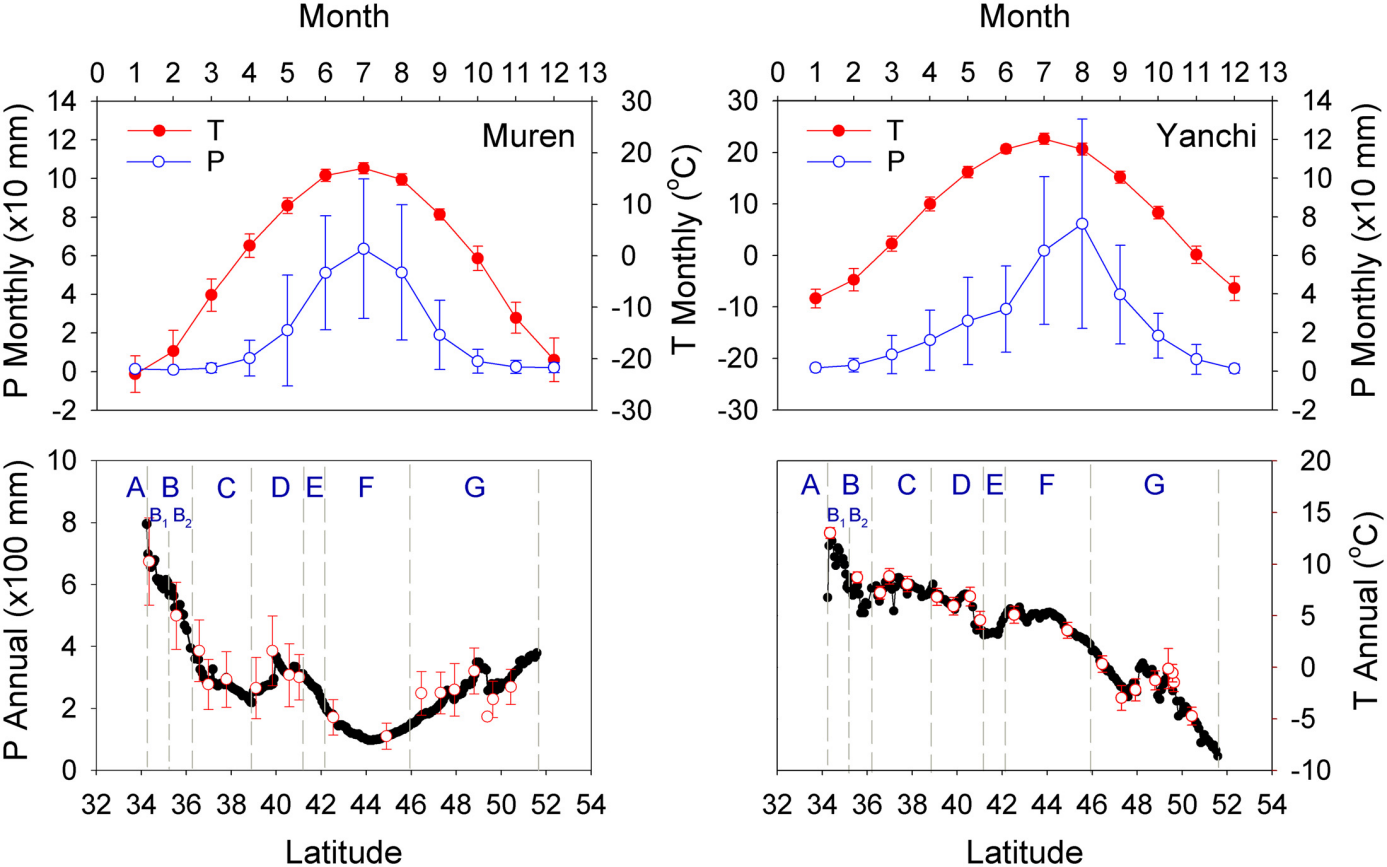
Variation of air temperature and precipitation in East Central Asia. Upper panels: Monthly changes of temperature and precipitation at the city Muren in north Mongolia and Yanchi in north China. Lower panels: Spatial changes in annual precipitation and temperature in the transect as interpolated (dots) and observed at meteorological stations (circles with error bars). The stations from south to north are Baoji, Pingliang, Haiyuan, Tongxin, Yanchi, Otog, Dongsheng, Baotou, Guyang, and Mandel, which are in China, and Buyant-Uhaa, Choir, Maanti, Ulaan Baatar, Bulgan, Hutag, Muren, and Hatgal that are in Mongolia.

East Central Asia consists primarily of north China and Mongolia. The climate changes from humid, through semi-humid and semi-arid to arid, and is characterized by extreme continentality (Fig 1). The winter is controlled by the Siberian/Mongolian High, which prevents maritime air-masses from moving inland, resulting in little precipitation [22]. The summer is subject to the pressure contrast between land and seas, the air-mass over the Arctic and Atlantic Oceans is driven southward, bringing precipitation as far south as north Mongolia, while the air-mass over the Pacific and Indian Oceans northward, generating the monsoon precipitation in south and east China [22, 23]. Either source of the moisture can hardly reach to the Gobi desert, where the meager precipitation is usually formed by strong convective movement of the atmosphere [24]. These climatic regimes result in the dry and wet seasons in East Central Asia with precipitation occurring predominantly in May-September, and a latitudinal distribution of precipitation roughly symmetrical about the Gobi desert (Fig 2).

**Fig 3:**
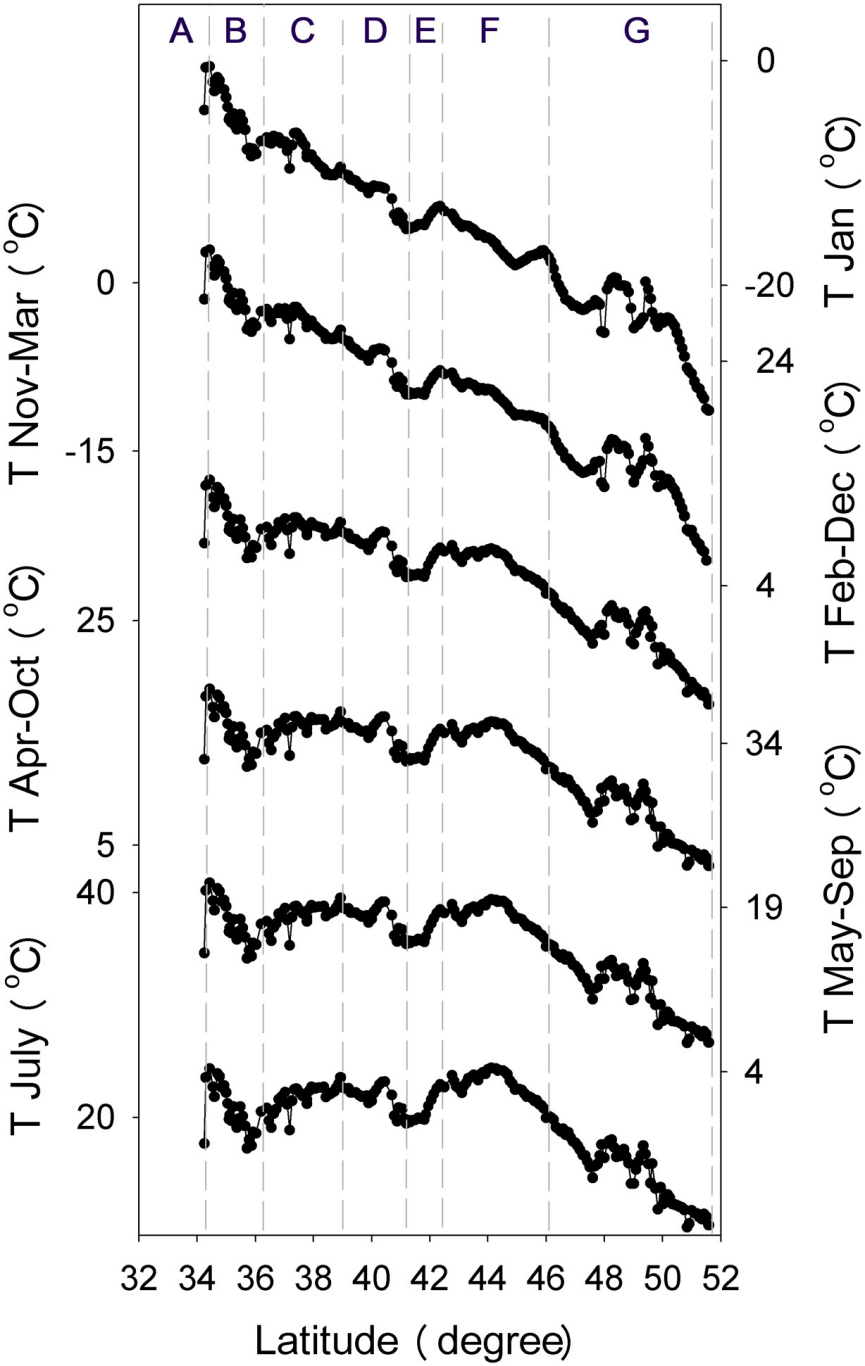
Temporal changes of the spatial distribution of air temperature in the transect. All the y-axes are in the same extent of scale.

Annual temperature changes in phase with precipitation in north Mongolia, but reaching its maximum one month earlier in north China (Fig 2). It decreases broadly from south to north due to latitudinal effect. This spatial trend, however, changes in time. As the season gets closer to mid-summer, it bulges increasingly up in section F due to summer heating in Gobi desert while drops progressively down in between section B and C for cooling of precipitation over Mount Liupanshan (Fig 3). Growing season starts in between April and May and ends after September [25–28], when temperature reaches up and drops down, respectively, about 10°C (Fig 2).

The effective precipitation, or water availability to the soil, which is the observed precipitation modified by evaporation, is indicated in this study by the De Martonne aridity index (AI), which is defined as *AI = P/(T+10)*, where P is the cumulative precipitation (mm) and T the average temperature (°C) in a specific period [29].

### Meteorological Data and Interpolation

The climatic data observed at the meteorological stations in the transect are from the China Meteorological Administration Bureau and the ‘‘Monthly Climatic Data of the World” prepared by the U.S. National Climatic Data Center in cooperation with the World Meteorological Organization. These data cover the years 1950–2000 for the stations in China and some in Mongolia.

Interpolation of the data was performed in 1 km grid cells by the thin plate smoothing spline surface fitting technique (ANUSPLIN version 4.36) [30, 31], which handles the effect of elevation by the STRM digital elevation model [32]. For the interpolation in China, the monthly temperature and precipitation were averaged from the observed data at 1814 stations, while for that in Mongolia, the data were generated from the FAO Local Climate Estimator that involved 74 stations [33]. As shown in Fig 2, the interpolated data agrees well with the records in the transect, especially in China.

### Sampling Transect

The sampling transect begins at 34°14'24"N, 106°55'30"E in Qinling Mountains, central China, and ends at 51°35'08"N, 100°45'49"E near the border of Mongolia and Russia, covering a distance 3020 km. It is divided into seven sections, denoted by A through to G, by geographical features (Fig 1). From south to north, the transect crossed a variety of zones of climate [24, 34], soil [34, 35] and vegetation that are distributed symmetrically across the Gobi desert [34, 36] (Table 1).

**Table 1:** Zones of climate, soil and vegetation crossed by the transect.

Transect	Climate	Soil	Vegetation
A	Sub-humid warm temperate	Acrisols	Deciduous forest
B	Sub-humid warm temperate	Calcisols	Deciduous forest
C	Sub-humid warm temperate	Arenosols	Steppe, Desert steppe
D	Sub-humid warm temperate	Solonchaks	Steppe, Desert steppe
E	Sub-humid medium temperate	Calcisols, Arenosols, Solonchaks	Steppe, Desert steppe
F	Sub-dry, dry and extremely-dry medium temperate	Calcisols	Desert, Desert steppe
G	Sub-humid cold temperate	Kastanozems	Steppe, Coniferous forest

### Sample Collection

Soil samples were collected in August 2002 at every 4'-5' latitude along the transect irrespective of longitude, corresponding to a distance about 15 km. The transect follows highways or other public driveways, the filed work along such a transect endangers no protected species, nor does harm to the land. Because of these natures, no specific permission was required for the sampling in either China or Mongolia. Since land use form is an important factor on SOC, the samples were collected only in uncultivated soil to avoid anthropogenic influence. In areas of intensive agriculture, such as section B1, the sample was taken in the ridge of fields. Totally, 210 sites were sampled.

Topsoil contains the bulk of organic matter [37] and is most subject to environmental changes [38]. Accordingly, all the samples were collected in top 10 cm in horizon A of the soil profile. The sampling was performed using a custom-made corer, which is 4 cm in diameter. The soil was cored every meter in a line of 5-meter long at each sampling site. six cores thus made resulted in a composite sample about 1.2 ~1.5 kg. At sites with an O-horizon such as in the forest of section G, the litters were removed first before sampling.

### Analytical Techniques

The experiments were done in the State Key Laboratory of Environmental Geochemistry. The samples were first air-dried at room temperature and separated manually with visible plant roots and gravels, and then treated subsequently as required by measurement of specific items.

#### Measurement of soil C and N content

The total C and N content was determined by a PE2400(II) element analyzer (Perkin Elmer). The samples were ground to pass through a standard 0.075 mm mesh sieve. Three aliquots of the soil (~ 2 mg each) were weighed and introduced to the instrument for the measurement. The standard deviation of replicate analysis is smaller than 0.3%. SOC and soil organic nitrogen(SON) were obtained by subtracting the respective inorganic components from the total values.

#### Measurement of soil inorganic C content

Soil inorganic C(SIC) was measured volumetrically by a Bernard type Calcimeter. An aliquot of the sample as treated above (1.25–5 g) was weighed and reacted with 10 mL of 10% v/v HCl in an Erlenmeyer flask. The CO_2_ developed from dissolution of the carbonate was measured volumetrically, the volume was used for calculating SIC content. The calibration was based on pure CaCO_3_, which was processed the same way as the samples. The standard deviation for 12 replicate analyses was 0.07%.

#### Measurement of NH4+-N content

NH_4_^+^-N was measured by the indophenol blue spectrophotometric method (China National Standard GB13580.11–92). An aliquot of the sample (~ 20 g) was weighed, transferred to a 200 mL Erlenmeyer flask, and added with 100 mL of NaCl solution (2 mol L^–1^). The mixture was shaken continuously for 1 hr on an end-over-end shaker, then left still over another hour and filtered. 5 mL of the solution was diluted with 5 mL of 2 M NaCl in a 50 mL colorimetric tube, and reacted for 1 hr with 5 mL phenol solution (0.1 mol L^–1^) and 5 mL alkaline sodium hypochlorite solution (0.0071 mol L^–1^). Then it was added with 1 mL EDTA solution to minimize possible interference of calcium and magnesium. The final solution was measured using an AquaMate Ultraviolet-Visible spectrophotometer (Thermo, USA) at wavelength 625 nm.

#### Measurement of NO_3_^−^-N content

NO_3_^−^-N was measured spectrophotometrically using the phenol disulfonic acid method (China National Standard GB/T 8538–1995). An aliquot of the sample (~ 20 g) as in the measurement of total C and N was weighed and extracted with CaSO_4_·2H_2_O solution (0.01 mol L^–1^) using the same procedure as described for NH_4_^+^-N measurement. A 15 mL aliquot of the filtered supernatant solution was mixed with 0.05 g CaCO_3_ and dried by evaporation at 60°C. After cooling down, the evaporite was reacted with 1 mL phenol disulfonic acid for 10 min, then added with 10 mL of pure water. The mixture was stirred until complete dissolution was achieved. After the solution was cooled down, 10 mL of NH_4_OH solution prepared by mixing the reagent (25%~28%) with water at 1:1 ratio was slowly added. The final solution was measured by an AquaMate Ultraviolet-Visible spectrophotometer at wavelength 420 nm.

#### Measurement of grain size distribution

Soil grain size was measured using a Malvern Mastersizer 2000. About 1.5 g air-dried soils was reacted successively with 20 cm^3^ of 10% H_2_O_2_ and 10 cm^3^ of 10% HCl on a heating plate (about 50°C) to remove organic matter and carbonate minerals, respectively [39]. The sample was then washed with distilled water, centrifuged and removed of the supernatant successively for a few times. Following this, it was added with 5.5 g/L sodium hexametaphosphate (NaPO_3_)_6_ as the dispersing agent. The mixture was subjected to ultrasonic oscillation for 5 minutes, and left still to react with the disperser for 24 hours before introducing to the instrument for measurement [40].

#### Measurement of soil pH

Soil pH was determined by a pHS-3CT pH meter (Jinnshine Photonics Technology Co., Ltd. Shanghai, China). An aliquot of 10 grams of the soils was mixed with 25 mL of pH-neutralized ultrapure water (Milli-Q Advantage, 18.2 MΩ) in a 50 mL beaker, the mixture was stirred for 1 minute, and the pH was measured in the aqueous solution [39].

### Statistical Analysis

All data analysis was done using the SPSS v.12 package (SPSS Inc., Chicago, Illinois). The correlation analysis was made between the climatic record and the average SOC at sampling sites near the meteorological stations. AI of different period was calculated using the average temperature and cumulative precipitation observed during the given temporal interval.

Path analysis was made between SOC and the environmental factors including elevation, mean grain size, pH, mineral N, C/N, as well as temperature and precipitation of various periods. To cross check with the correlation analysis, the data used in the path analysis were interpolated ones based on the sampling site instead of the means on the meteorological stations.

## Results

### Soil Organic Carbon

**Fig 4:**
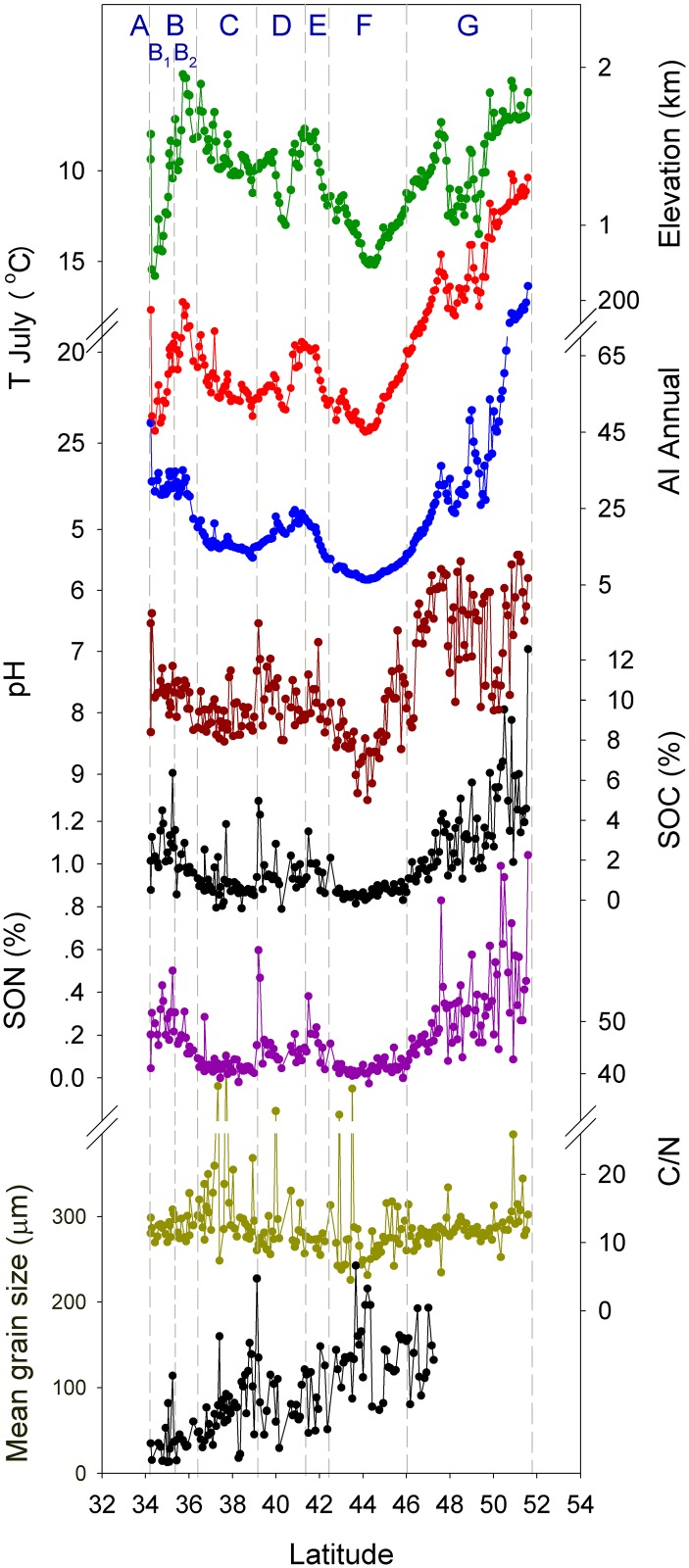
Spatial change of elevation, climate, SOC and other soil properties in the transect. Note the inversion scale of temperature and pH.

SOC content is about 2.5%(w/w) in section B1, decreasing northwards to 1.8% in section B2, and to 0.5% in section C. It increases to about 1.3% in sections D and E, and drops to 0.3% in section F. Further north, SOC increases from 1% to about 10%, averaging 5%, throughout section G (Fig 4). SOC in section B1 and section D-E is 5 and 2 times, respectively, as high as in the adjacent section C in north China. The difference reached about 17 times between section G and F.

**Fig 5:**
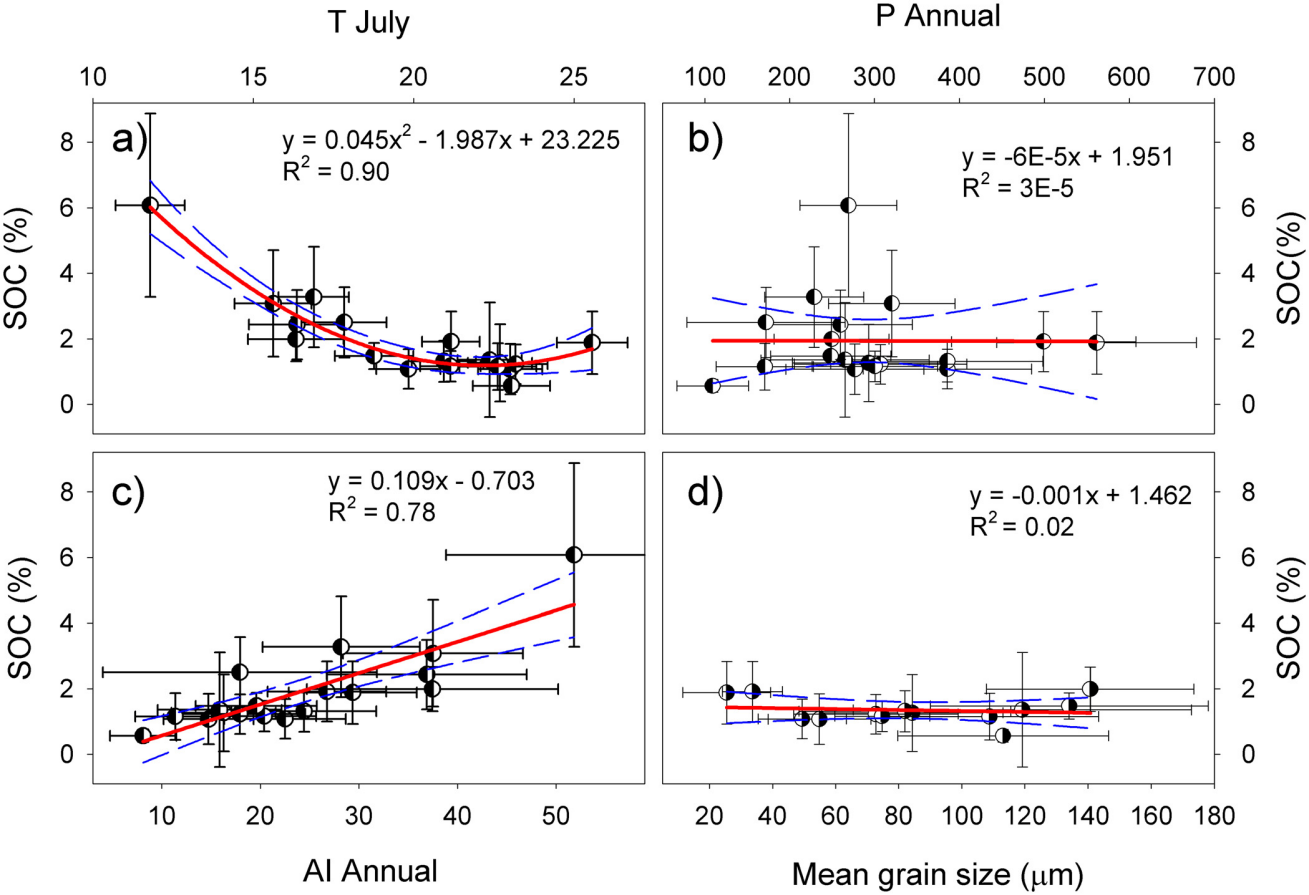
Relationship of SOC content with July temperature (a), annual precipitation (b), annual aridity index (c) and the mean grain size of soil particles (d). The error bars are 2σ standard deviations. The dashed lines bracket the 95% confidence band of the regression analysis.

**Fig 6:**
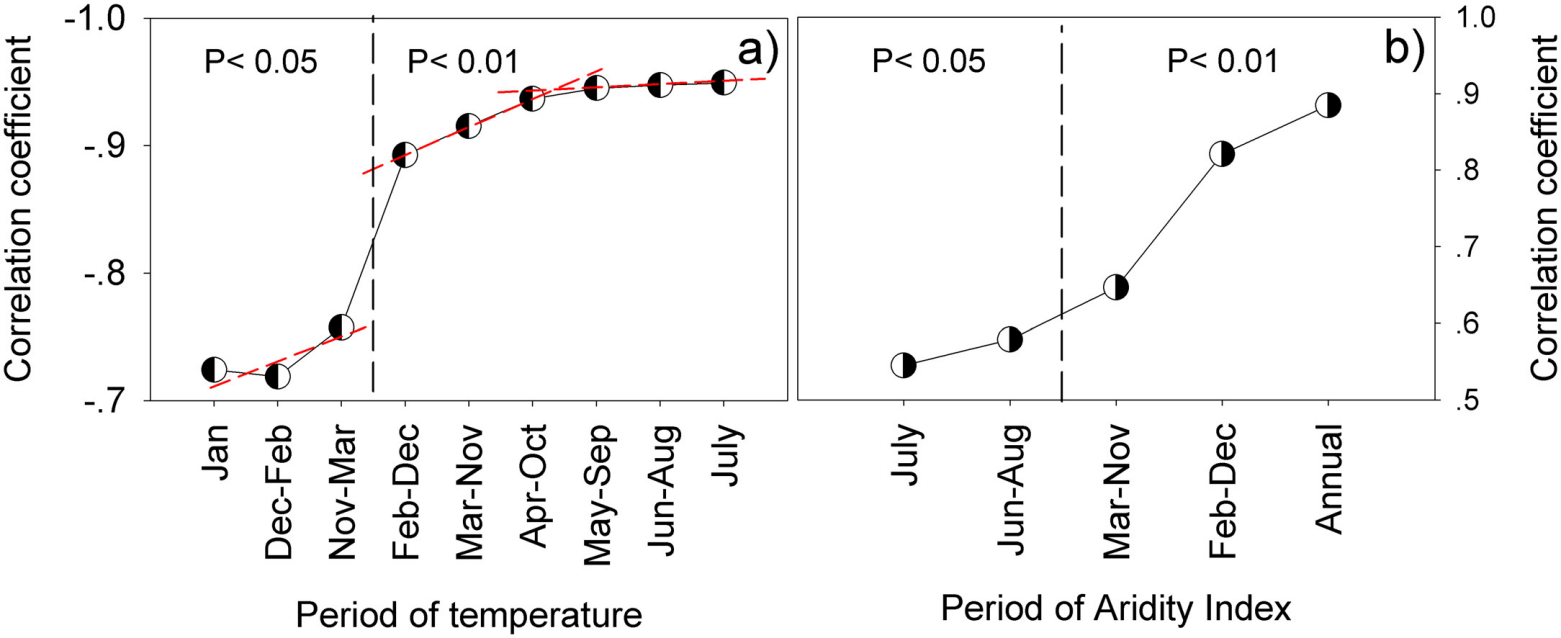
Correlation of SOC content with temperature (a) and AI (b) at different periods of a year. The vertical dashed line divides the confidence levels of P < 0.05 to P < 0.01.

SOC decreases with temperature in various periods of a year, especially in July, following a quadratic equation (Fig 5a). The trend slowed down as the temperature reached about 20°C, corroborating the change of equilibrium carbon content with temperature as predicted by numeric models [41]. The relationship weakens as temperature goes down in other periods of a year, with a small yet quicker drop in between May-September and April-October (Fig 6a). The weakened relationship indicated reduced dynamics of the carbon pool while the faster drop the slowed carbon gain due to cease of plant growth. As the growing season, May-September is a period for carbon input via plant growth, root growth in particular [42, 43]. The coexistence of carbon gain and loss during this time, both affected by temperature, brings strong dynamics to the carbon pool, and thus a strong correlation with temperature. With cessation of the growth in February, March, April, October, November and December, which were taken into account successively in the period April-October, March-November and February-December, only the process of decomposition remains in the soil. This change turned the reduction of correlation to a faster trend. The change is nevertheless small, suggesting that the effect of temperature on the carbon gain is overwhelmed by that on carbon loss, and therefore ends up with the negative relationship with SOC content. The correlation dropped dramatically in winter months because even soil respiration becomes small, particularly in January [44].

This mechanism concurs with the results of path analysis, which examines the causal linkage between statistical variables [30]. As shown in Table 2, the total effect of temperature is all negative with the absolute value decreasing from July through the various periods to January, confirming that increase of temperature reduces SOC despite primary production occurs in the season of high temperature. The effect is taken primarily in direct ways as indicated by the large direct effect. The indirect effect is small and works differently on SOC between winter and summer months. It is positive and mainly through precipitation in the winter half year, contributing to carbon accumulation, while negative and about equally by various factors in the summer months, causing decay of the organic matter.

**Table 2:** Path analysis on the effect of P, T and other factors on variation of SOC.

Period	Factor	Total effect	Direct effect	Indirect effect via						R^2^
				total	P	T	NH_4_^+^	elev.	pH	
July	P	0.54	0.20	0.33		0.31	0.08	-0.06		0.622*
	T	-0.73	-0.62	-0.11	-0.10		-0.09	0.08		
	NH_4_^+^	0.51	0.23	0.28	0.07	0.24		-0.04		
	Elev.	0.41	-0.13	0.54	0.09	0.39	0.07			
Jun-Aug	P	0.48	0.23	0.25		0.25	0.07	-0.07		0.628**
	T	-0.72	-0.64	-0.08	-0.09		-0.09	0.10		
	NH_4_^+^	0.51	0.23	0.28	0.07	0.25		-0.04		
	Elev.	0.41	-0.16	0.57	0.10	0.40	0.06			
May-Sep	P	0.39	0.27	0.12		0.11	0.06	-0.05		0.624*
	T	-0.70	-0.64	-0.07	-0.05		-0.09	0.07		
	NH_4_^+^	0.51	0.23	0.28	0.07	0.24		-0.03		
	Elev.	0.41	-0.12	0.53	0.11	0.36	0.06			
Apr-Oct	P	0.35	0.30	0.05		0.00	0.05			0.618**
	T	-0.67	-0.59	-0.08	0.00		-0.08			
	NH_4_^+^	0.51	0.23	0.28	0.07	0.21				
Mar-Nov	P	0.34	0.36	-0.03		-0.08	0.05			0.617**
	T	-0.63	-0.60	-0.03	0.05		-0.08			
	NH_4_^+^	0.51	0.22	0.29	0.08	0.21				
Feb-Dec	P	0.33	0.40	-0.07		-0.13	0.05		0.01	0.613**
	T	-0.59	-0.58	-0.01	0.09		-0.07		-0.03	
	NH_4_^+^	0.51	0.21	0.31	0.09	0.19			0.03	
	pH	-0.62	-0.05	-0.56	-0.08	-0.38	-0.11			
Annual	P	0.33	0.42	-0.09		-0.15	0.05		0.01	0.612**
	T	-0.57	-0.58	0.01	0.11		-0.06		-0.04	
	NH_4_^+^	0.51	0.20	0.31	0.10	0.18			0.03	
	pH	-0.62	-0.06	-0.56	-0.08	-0.37	-0.11			
Nov-Mar	P	0.13	0.49	-0.36		-0.40	0.02	0.02	0.00	0.586**
	T	-0.46	-0.63	0.17	0.31		-0.05	-0.02	-0.07	
	NH_4_^+^	0.51	0.19	0.32	0.05	0.16		0.04	0.06	
	Elev.	0.41	0.15	0.26	0.08	0.09	0.05		0.04	
	pH	-0.62	-0.12	-0.50	0.01	-0.36	-0.10	-0.05		
Dec-Feb	P	0.15	0.41	-0.26		-0.32	0.03	0.03	0.00	0.551**
	T	-0.44	-0.53	0.09	0.25		-0.05	-0.02	-0.09	
	NH_4_^+^	0.51	0.19	0.32	0.06	0.13		0.05	0.08	
	Elev.	0.41	0.17	0.25	0.08	0.06	0.05		0.05	
	pH	-0.62	-0.16	-0.45	-0.01	-0.29	-0.10	-0.06		
Jan	P	0.25	0.40	-0.15		-0.23	0.03	0.04	0.01	0.564**
	T	-0.42	-0.46	0.03	0.20		-0.04	-0.01	-0.11	
	NH_4_^+^	0.51	0.19	0.33	0.07	0.11		0.04	0.11	
	Elev.	0.41	0.14	0.27	0.11	0.04	0.05		0.07	
	pH	-0.62	-0.21	-0.41	-0.02	-0.24	-0.10	-0.05		

*P<0.05;

**P<0.01

Precipitation is important in plant growth and, to a lesser degree than temperature, in decomposition of SOC as reported in “precipitation pulse”, a phenomenon of abrupt increase in soil respiration following soil’s wetting [45–47]. The observed precipitation, however, bears no significant correlation with SOC (Fig 5b) due to change by evaporation. Because of this, we inferred the role of precipitation by studying the relationship between SOC and AI. The results show that the variation of AI correlates positively with SOC (Fig 5c), explaining as much as 88% of SOC variability compared to 81% by July temperature alone. The correlation weakens from the annual basis through to July (Fig 6b), suggesting even the minor precipitation in the winter month makes a difference.

Path analysis proved that precipitation indeed has a positive effect on SOC with the total effect decreasing from summer to winter (Table 2). It works primarily through temperature in the growing months when the indirect effect even surpassed the direct one, stressing the importance of temperature in the effect of precipitation. This indirect effect quickly dropped and became negative after April-October, suggessting the change of its role from growth to decay of SOC after cessation of plant growth.

### C:N of Soil Organic Matter

SON is lower than SOC by about one order of magnitude, its spatial variation and even most local fluctuations, however, are similar to SOC (P < 0.01) (Fig 4). Clearly, there is a strong association between SOC and SON. As a result, the molar ratio C:N, which depicts the association quantitatively, is rather consistent in comparison to the large variations of the content. It averages about 12 in most transects except section C, suggesting that the soil organic matter maintains a consistent stoichiometry in C and N in most regions of East Central Asia. Interestingly, a generally high C:N about 17 was observed in section C, which is located in the agro-pastoral transitional zone in Chinese history. The reason for the high C:N remains to be studied.

In reviewing the literatures on C and N in surface soils, Cleveland and Liptzin found that globally, SOC shows a C:N about 14 despite high biological diversity, structural complexity and spatial heterogeneity of soils [48]. The same is also found for the surface soils in China [49]. Compared with these observations, C:N is rather low in East Central Asia. Nevertheless, it is in agreement with the mean for uncultivated soils in temperate regions [50], suggesting that land use might be a factor in C:N of SOC.

### Soil pH

pH is about 8.0 in north China and south Mongolia while about 6.4 in north Mongolia. The highest alkalinity occurs in the Gobi desert followed in the region adjacent to the Mu Us sandy land. The general alkalinity of north China is interrupted by lowered pH in the Qinling Mountains, Yellow river valley and Yinshan Mountains while the acidity in north Mongolia interrupted by the high alkalinity around 50°N (Fig 4).

The regional variation of soil acidity is negatively correlated with annual AI (P<0.01), and the acidic soils in north China are either located in mountains or river valleys where water availability is high and thus leaching of ions such as H^+^, Al^3+^, Ca^2+^, Mg^2+^, K^+^, and Na^+^ is strong, all confirming the importance of leaching vs evaporation in the variation of pH [51]. Parent material of the soil does matter, the effect, however, is only apparent about 50°N, where the “liming” effect of carbonate bedrocks resulted in the high alkalinity.

pH affects SOC, but its effect only passed the significant test in the winter months (P<0.01). It works negatively and predominantly in indirect ways via temperature (Table 2).

### Soil Grain Size

Grain size distribution quantifies the content of clay minerals, silt and other larger particles of soil, indicating soil texture. This study analyzed the grain size of the soils south of section G and found that the texture is not a major factor in the variation of SOC. The mean grain size reduces in fluctuation from the Gobi desert to the Qinling Mountains (Fig 4). It is consistent with the provenance of the loess in Central Asia, which was deposited in order of grain size as transported southeastwards by wind [52]. The soils developed in the deposition while preserved the particle distribution. But it increased slightly in the northern section C and southern central section F, indicating that desiccation in the Mu Us sandy land and the Gobi desert later on coarsened the soil texture. These modifications nonetheless did not change the general trend, leaving the spatial variation far different from SOC (Figs 4 and 5d). Path analysis showed that the mean grain size failed to pass the significant test at the 0.05 level when adopted into the models, confirming its negligible role in the variation of SOC.

### Soil Mineral Nitrogen

**Fig 7:**
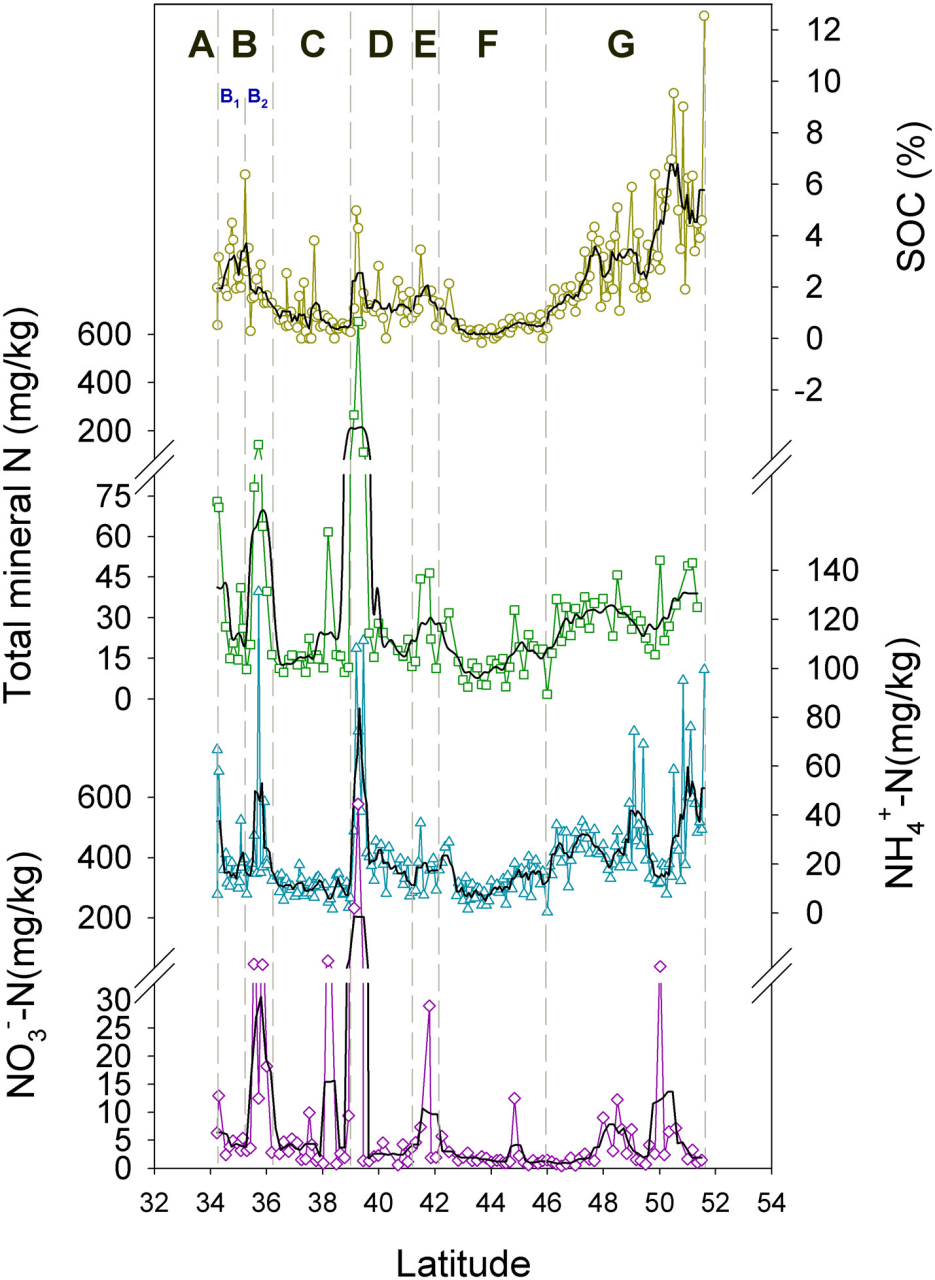
Soil mineral N in comparison to SOC. Only the total N that includes NO_3_-N was shown in the graph.

Soil mineral N, i.e. NH_4_^+^-N and NO_3_^-^N, show a basic variation and some extraordinarily high spikes on it (Fig 7). The basic NH_4_^+^-N is about 20 mg/kg with the spikes over 60 mg/kg in the Dustin River valley, the northern edge of the loess plateau, the Qinling Mountains and a few places in north Mongolia. These areas are all of intensive stock farming, suggesting that animal excreta are responsible for the spiking NH_4_^+^-N. The basic content of NO_3_^−^-N is about 2 mg/kg, which is 10 times lower than NH_4_^+^-N. The spikes of NO_3_^−^-N either coincides with NH_4_^+^-N, suggesting the animal provenance, or occurs in fluvial sediments as a result of deposition of water-dissolved NO_3_^-^.

NH_4_^+^-N changes with SOC significantly (P<0.01), and affects SOC year round as indicated in path analysis. It increases SOC primarily through temperature, especially in the growing months. NO_3_^−^-N shows no correlation with SOC due probably to modification of water leaching. Despite the latter, the variation of total mineral N still correlates significantly with SOC (P<0.05).

### Topography

Topography changes dramatically in the transect as shown by the elevation (Fig 4) and correlates significantly with SOC (P<0.01). It affects the spatial variation of climate, especially air temperature in the summer season (Fig 3). As a result, the effect of topography is performed primarily via temperature (Table 2). The indirect effect is helpful for SOC accumulation, and is much stronger in the growing months than in winter ones. Although relatively small, the direct effect differs on the season. It contributes to SOC growth during winter but to its decomposition in the growing season. Despite the difference, the total effect is positive, making SOC increase with elevation.

## Discussion

Globally, SOC was found to be correlated negatively with temperature while positively with precipitation [21]. Our results in East Central Asia indicated further that the negative relationship with temperature was indeed a result of positive effect of temperature on decomposition of SOC, and that direct effect of temperature on the decomposition occurs through a year although the indirect effect via precipitation contributes to the carbon accumulation in the winter season.

The relative sensitivity of net primary productivity and decomposition of soil organic matter to change of temperature is critical for understanding the feedback of soil carbon pool to the climate change [53]. Our results indicated that the effect of temperature on the decomposition is much larger than on the carbon input by primary production.

Our results also confirmed precipitation contributes to SOC accumulation, its effect, however, is relatively small and depends strongly on temperature, making the Annual AI correlated significantly with SOC.

The dominant control of temperature on SOC as found in our study is the same as observed in Siberia in the high latitude [17]. The identity, however, lies in the effective precipitation rather than temperature as the important factor when compared to the tropical Africa in the low latitude [20]. It seems that a shift of most effective climatic factors occurs in the mid-latitude. Soil texture is an important factor in variation of SOC in both Siberia [18] and tropic Africa [20], and even in the loess plateau [19], which is included in our studying area, it is not true, however, in the East Central Asia. We believe this indicated the importance of spatial scale in the constraints of SOC.

pH appeared in many studies as correlated with SOC, and thus was even adopted as a factor for estimating the carbon inventories in an empirical model [54]. Our study, however, indicated its effect occurs mainly in the winter months.

As the predominant source of SOC, vegetation may be regarded as an important factor on the variation of SOC given that it changes dramatically in the transect (Table 1) and that different plant can differ in carbon input [37]. Vegetation types distribute roughly in symmetry about the desert vegetation zone in East Central Asia, following the spatial distribution of annual AI. It confirms the role of climate in the spatial distribution of vegetation [34, 36]. Accordingly, we believe, albeit unable to quantify in the path analysis, that the varied carbon input of plants is likely responsible for some of the local fluctuations of SOC, and that the change in vegetation distribution should have played a role in the variation of SOC, particularly in indirect ways through climatic factors. The same reasoning also applies to the notion of vegetation as a factor on variation of pH [55].

Soil type may be regarded as well as an important factor since it can differ significantly in SOC content. It is worth noting, however, that development of a soil type depends on climate [56], which is corroborated by the soil distribution in the East Central Asia (Table 1). Kastanozems, for example, has much higher SOC than Calcisols, the reason, however, lies in that the former develops in the cold temperate climate while the latter in the dry temperate. The effect of soil type is, therefore, supposed to work indirectly through climate factors.

Airborne N was reported in many studies as affecting vegetation species and productivity [57, 58] and thus on SOC content. Its contribution is unable to be isolated from other mineral N because the deposition flux is unknown in the transect. A simple estimation, however, indicated it should be trivial. Assuming the deposition flux as 20 kg N ha(-1) yr(-1) as observed in China in recent decade [59] and a soil bulk density 1.0 g/cm(-3), the annual deposition should make up a 2.0x10^-3^ mg/kg in the top 10 cm of soil. Given that the flux was even lower before, being 13 kg N ha(-1) yr(-1) in 1960s, for instance, the total airborne N accumulated should be less than 0.2 mg/kg over the past century, which is even one order of magnitude lower than NO_3_^−^-N. This estimation agrees with recent findings that nitrogen deposition has a minor impact on the global carbon cycle [60].

Despite that the factors of vegetation and soil type were not included in the path analysis, the results still achieved R^2^ as large as 0.62 in the model testing, especially for the summer months (Table 2). The factors adopted in the regression model explained about 79% of the SOC changes, and thus represented the major constraints on the dynamics of SOC in East Central Asia.

Air temperature increased about 0.8°C in East Central Asia since 1970s [61] in comparison to 0.6°C in the northern hemisphere [62], while annual precipitation remains relatively invariant despite some areas with slight increase [63]. Although the warming occurs primarily in winter half year, when both vegetation growth, soil respiration and the effect of climate are minimum, it certainly reduces soil annual AI by enhancing evaporation. If this regime of climate change remains unchanged, then by the linear model of SOC with AI (Fig 5c), a 0.8°C warming in East Central Asia will reduce the SOC content by about 10% in north China, 6% in the Gobi desert and 34% in north Mongolia. The higher the water availability now, the stronger the carbon loss in the future. This process, however, may take time, for as long as centuries [41].

## Conclusions

SOC content changes dramatically in East Central Asia. The regional difference is as high as 5 times in north China while 17 times in Mongolia. Despite the content, C:N is rather consistent about 12, indicating a similar stoichiometry in the organic matters. Temperature is the dominant factor in the variation of SOC with a direct effect on decay of SOC the year round, especially in the summer months, and an indirect effect, mainly via precipitation, on the growth of SOC in the winter months although the latter is much smaller. Precipitation helps build up the SOC stock, a large part of the effect, however, is taken via temperature, which leads to SOC growth in the growing months while to its break-down in the other ones. This makes aridity index correlated significantly with SOC. NH_4_^+^-N and topography also help with accumulation of SOC, their effect is taken primarily via climatic factors, temperature in particular. pH correlates significantly with SOC as does temperature and aridity index, its effect, however, only occurs in the winter months, helping decompose SOC primarily through temperature. Altogether, these factors explained as much as 79% of the variation of SOC, especially in the summer months, and represented the major constraints on the SOC stock. Changes in vegetation, soil type, which are all at the control of climate, may contribute to the change of SOC, especially in indirect ways, their effects, however, remain to be proved. Soil texture does not constitute a factor in the variation of SOC. These findings suggested that, with temperature as the dominant factor, the recent global warming should have been reducing SOC stock in the terrestrial ecosystem of the mid-latitude.
